# A New Treatment for Impetigo Contagiosa

**Published:** 1910-10-01

**Authors:** E. M. Magill


### A NEW TREATMENT FOR IMPETIGO CONTAGIOSA.

By E. M. MAGILL, L.S.A.

As a student I remember well the long relays of impetigo contagiosas which frequented the out-patient department, and which recovered with more or less expedition according to the precision with which the directions accompanying the ointment were carried out. But I was particularly impressed at that time with the idea of a treatment recommended by the skin specialist for the use of those with leisure and patience, attributes not always possessed by the general practitioner. This treatment consisted in the application of a continuous stream of warm normal saline solution flowing over the affected parts for as long as possible—twelve hours or so at a stretch. The surgeon remarked that the drawbacks of the treatment were sufficiently obvious, but that it would at all events enable the child to become sufficiently presentable to go to a party the next day. Some time ago I had the good fortune to be able to put the idea into practice, with the most satisfactory results. The patient, a child of two years, was in an extremely bad condition. She had been suffering for more than six months with a severe eruption round the mouth and nose. She had, during the whole of this period, been receiving medical treatment with ointments, but instead of improving had been getting gradually worse. Indeed, the eruption had now become so bad that the mother felt that she could not take the child out in the street. The child's appearance caused extreme distress to the parents, who were clean and respectable people, and they were entertaining disturbing ideas with regard to the significance of the rash.

When I first saw the case it was a typical impetigo contagiosa; there were thick masses of yellow crusts with inflamed bases, some dry, some moist with pus, all around the mouth and nose, extending over the cheeks, forehead, and on to the neck. There was really very little healthy skin visible, so general had the eruption become. A good many small patches were also to be seen on the child's hands and arms.

I suggested the saline treatment, explaining that it would be very tedious and difficult with so small a child. The mother was most anxious to undertake it, however, as she had already tried ointments for so long in vain.

A large quantity of warm normal saline solution was prepared and kept in readiness, and a douche can with a long tube, stop-cock, and fine nozzle was hung at a convenient height. The baby, swathed in mackintoshes, had to be held in turns by mother, nurse, or friend, as comfortably as possible, so that the stream of normal saline from the douche can could be directed on to the various affected parts of the face. The baby's relations assisted in carrying out the treatment most efficiently, and the continuous bathing of the sores was kept up, with very few interruptions, for eight hours on end. The child was extremely good on the whole, apparently finding the warmth of the water comforting.

On leaving off for the evening there was not a scab left on the face, nor any pus, while in place of the old sores there was a tender, pink epithelium. The changed appearance was most marked. Before leaving I dressed all the infected places on the child's arms and hands with unguentum hydrarg. ammon. 5 grs. to the ounce, after removing the scabs, carefully covering the sores in order to prevent any re-infection of the face.

I was very anxious to see if any scabs or pus would re-form before the morning, but was very gratified to find the face beautifully clear; the situation of the old sores was still indicated by the redness of the new epithelium, but the appearance of the child was no longer repulsive.

Two days later the parents took the child out to a public entertainment, the first time for many months; its appearance was quite unnoticeable.

I have never known so extensive and old-standing a case cured as quickly by the mere application of ointment, though for the majority of patients the latter treatment is the more convenient.

